# Prevalence and Predictors of Alcohol Use among School-Going Adolescents in Panama: A Population-Based Cross-Sectional Study

**DOI:** 10.3390/children10050891

**Published:** 2023-05-16

**Authors:** Paul Obeng, Francis Sambah, Jacob Owusu Sarfo, Medina Srem-Sai, Newton Isaac Gbordzoe, Richmond Stephen Sorkpor, John Elvis Hagan

**Affiliations:** 1 Department of Health, Physical Education and Recreation, University of Cape Coast, Cape Coast PMB TF0494, Ghana; 2 Department of Health, Physical Education, Recreation and Sports, University of Education, Winneba P.O. Box 25, Ghana; 3 School of Public Health, University of Ghana, Legon P.O. Box LG 13, Ghana; 4 Neurocognition and Action-Biomechanics-Research Group, Faculty of Psychology and Sports Science, Bielefeld University, Postfach 10 01 31, 33501 Bielefeld, Germany

**Keywords:** adolescents, alcohol use, Panama, predictors, prevalence, substance use

## Abstract

This study investigated the prevalence and predictors of alcohol use among school-going-age adolescents in Panama. Using a national school-based cross-sectional survey, data from a proportionate sample of school-going adolescents aged 13–17 years were obtained from the 2018 Panama Global School-based Student Health Survey [GSHS]. Data were analysed with a Pearson’s Chi-square test and weighted binary logistic regression. The results were reported with their corresponding adjusted odds ratio (AOR) at a 95% confidence interval (CI) and level of significance set at *p* < 0.05. The prevalence of alcohol use among adolescents in Panama was 30.6%. The odds of alcohol use were lower among adolescents in a lower grade than those in upper grades, and lower in those who did not eat from a restaurant than those who ate from a restaurant. Further, the likelihood of alcohol use was significantly high among those who engaged in physical fights, were seriously injured, were mostly worried, and whose parents used any form of tobacco. Other results showed that the odds of alcohol use were high among sedentary respondents, those who had multiple sexual partners and those who used amphetamines. Based on the present findings, a collaborative approach (i.e., stakeholders- the Ministry of Social Development and the Ministry of Education- community–individual levels) towards the development and adherence of appropriate interventions aimed at reducing alcohol use is required in Panama. Specific preventive interventions would be fundamental in promoting a positive school climate to help reduce adolescents’ alcohol use and, perhaps, other anti-social behaviours (e.g., physical fights and bullying).

## 1. Introduction

Alcohol use in adolescent years has been identified as a major public health issue that poses severe health and social consequences on the well-being of people [1,2]. About 2.8 million deaths recorded in 2016 were linked to alcohol use globally, which relates to 6.8% of the age-appropriate deaths in males and 2.2% in females [2]. Concerning the total burden of disease, alcohol use is rated as the seventh prominent risk factor for premature death and disabilities, including the global burden of diseases [3]. The public health burden of alcohol use accounts for approximately 5.1% of the global disease burden [4].

From the perspective of the problem behaviour theory, adolescents who indulge in socially undesirable behaviours will be more prone to engage in different dangerous behaviours. These behaviours possibly co-occur, leading to violent acts, physical fights, injuries and behaviours that are sexual [5,6]. Alcohol use (i.e., early initiation, frequent consumption and drunkenness) among the adolescent population has many health implications (i.e., negative psychological, social and physical health consequences), including road accidents, attempted suicides, injuries, physical and sexual assaults and increased in risky sexual behaviours [7,8,9]. According to Silveri [10], early usage of alcohol can lead to lifetime dependence because using alcohol before the age of 15 is linked with 38% of lifelong dependency. Regular alcohol use in early life can lead to addiction, which is also connected with intellectual and brain deformities and poor decision-making abilities even in adulthood [11,12]. Mutumba and Schulenberg [13] indicated that alcohol use adds to almost half of the illnesses that occur among young people between the ages of 10 and 24 years [14,15,16] and also impedes their capability to effectively transition through the developmental processes linking adulthood [17].

Available evidence suggests variations in alcohol use across different geographical settings worldwide [18]. Specifically, alcohol use prevalence by 2014 significantly varied across the European region, ranging from 2% to 26% among females and 3% to 33% among males, with higher variation among males and the highest levels in Central–Eastern European and Southern Europe/Mediterranean subregions and lowest in the Nordic countries [4]. Similar patterns and prevalence of alcohol use have been reported in the Latin America and Caribbean region. Statistics indicate that the past two decades have seen an upsurge in alcohol consumption, with a prevalence rate of ≥30% across some countries such as Bolivia, Grenada, Panama and Trinidad and Tobago, as well as in El Salvador, Guatemala and Uruguay, including higher rates of heavy episodic drinking, explained as five or more drinks per occasion for males and four or more drinks per occasion for females, which is twice the global average [19,20]. According to the Pan American Health Organization [20], alcohol use remains a top-six risk factor for morbidity in the region, with males more likely to suffer alcohol-related injuries than females. However, the gap has been bridged because of higher rates of alcohol-related injuries among females in the region.

Alcohol use develops over time and is primarily associated with a dynamic interplay between a myriad of individual, familial and socio-environmental factors, with gender, age, socio-economic status, social experiences, cultural norms and other social circumstances all playing a significant interactive role [4]. Existing research has shown that alcohol-related behaviours are characteristically more frequent among males than females [21], though there seems to be ample evidence for increasing gender convergence among adolescents attributable to changing gender role orientation in many countries [4,19,22]. Drinking culture has also been found to be more common among adolescents with increasing age, although males reportedly use more alcohol than females, contributing substantially to peer violence [10,23]. Further, other demographics (grade level), personal (not eating at a restaurant, being truant) and psychosocial (being a physically attacked victim, engaging in physical fights, experiencing a serious injury, suicidal ideation, suicidal plan, attempting suicide, outside-school bullied victims, cyberbullied victims, parental tobacco use, living a sedentary lifestyle, having multiple sexual partners, using amphetamines, feeling lonely and worried) have been established as significant predictors of alcohol use among adolescents in schools [18,24,25,26]. Recently, Sarfo et al. [27] found that current use of tobacco and other drugs, including alcohol, smoking cigarettes, bullying, loneliness, worrying and having close friends, were associated with suicidal behaviours among adolescents in Saint Vincent and the Grenadines.

Other research has documented the relationship between family socio-economic background and alcohol use in adolescents. Socio-economic disparities in adolescents’ alcohol use are stronger in poorer countries and those with high-income disparity [28]. Similarly, a history of alcohol and other substance use has been revealed as a major risk factor for adolescents’ alcohol use [29,30,31]. Likewise, parents or other family members who have challenges with alcohol and other substance use may model substance use, increase opportunities for alcohol use by having these substances available, or fail to monitor adolescents’ behaviour, thus increasing the likelihood of adolescents’ alcohol use [29,30,31].

Panama forms part of the changes in the Central America region with the continuous insurgence of drug trafficking and violence attributable to drug use and abuse among the adolescent population in this region [19,32,33]. According to Palma-Álvarez et al. [33], the distribution of alcohol consumption among adolescents 15 years or older, with beer and spirits accounting for 77% and 19% of alcohol usage in the country. Even though the legal age for drinking alcohol in Panama is 18 years, there is a gap in law enforcement. The government of Panama has made several policy interventions in the past and in recent times to control age-appropriate purchasing, possession and use of alcohol [2]. However, the country lacks a national policy or action plan [2]. For instance, the government has a national legal minimum age for off-premises sales of alcoholic beverages such as beer, wine and spirits, as well as legally required health warning labels on alcohol advertisements and sales to young people [2]. In addition, the government has further decided by policy to increase alcohol taxes to help address some of the risk factors, such as cancer. Despite these sound policies, enforcement has been lacking on the part of law enforcers, and that may be undermining the already established structures meant to protect adolescents’ influence, use and abuse of alcohol and other substances [2].

Many adolescents under the age of 18 years use alcohol on the blind side of the law, leading to social and behavioural problems and long-term health defects among the young Panamanian population. Panama’s drug surveillance system revealed an upsurge in drug use and drug-related problems among young individuals who seek emergency care medically, those who seek treatment for abusing substances, including alcohol, and inmates who reside in rehabilitation centres [33]. The high prevalence rate of alcohol use among junior school grade level students calls for improving primary prevention actions to address this public health challenge in the country through current research that may help guide health education and promote interventions. Findings from this research could help Panama in the progressive achievement of Sustainable Development Goals 3 and 4, which seek to promote healthy lives and quality education for adolescents globally by 2030 [34]. Thus, given the sparse empirical evidence in Panama, we investigated the current prevalence and predictors of alcohol behaviour among school-going adolescents in the country. It was hypothesised that the selected socio-demographic (age, grade, sex), personal (not eating at restaurants, sedentary lifestyle and truancy), drugs and substance use (amphetamine use), and psychosocial factors (physical fight; physical attack; suicidal ideation, plan and attempt; bullied away from school, cyberbullied, parent’s/guardian’s use of tobacco, understanding parents, parental checks on completeness of assignment, multiple sexual partners, parent’s/guardian’s knowledge of what adolescent does with free time, loneliness, worry) would significantly be associated with alcohol use among in-school adolescents.

## 2. Materials and Methods

### 2.1. Study Design and Setting

This study is a national school-based cross-sectional survey that collected data from school-aged adolescents in Panama. Panama is a country on the isthmus that links Central and South America. Panama is one of the WHO countries interested in understanding the behavioural risk factors and protective factors associated with morbidities and mortalities among in-school adolescents aged 13–17 years. The data were obtained from the 2018 Panama Global School-based Student Health Survey [GSHS]; the repository link is as follows: https://extranet.who.int/ncdsmicrodata/index.php/catalog/879 (accessed on 22 August 2022). The WHO carried out the GSHS in collaboration with the Centers for Disease Control and Prevention (CDC), Panama’s Ministry of Health (MoH) and the Ministry of Education (MoE). A structured, self-administered questionnaire with closed-ended questions was used to collect data. The study’s instruments were pilot-tested, and the instrument’s reliability and validity were determined before data collection began.

### 2.2. Ethical Consideration

The Panama MoH and MoE Institutional Review Boards provided ethical clearance (approved on January 1 2018) before the start of the main study. With the ethical clearance certificate in hand, entry permission was sought from the Panama MoE and the principals of all participating schools. In addition, child assent was obtained from participants under the age of 18, along with parental consent from their parents. Furthermore, informed consent was obtained from participants aged 18 and up through both written and verbal means.

### 2.3. Sampling

Data was gathered by randomly selecting students from Panama’s eighth to twelfth grades. In terms of sampling participants, the procedure was divided into two stages. First, schools were chosen with a probability proportional to enrollment size. Second, classes were chosen at random, and all students in those classes were eligible to participate. The overall response rate was 59%, with 94% from the school and 63% from the students. Overall, a total sample of 2914 participants was included in the study.

### 2.4. Study Variables

Our study variables were selected based on our conceptual framework that multiple factors caused the current use of alcohol among school-aged adolescents. The dependent variable was “current use of alcohol” among school-aged adolescents, defined as “whether the participant currently drinks alcohol or not”. The respondents were given the choice of “Yes or No”. Participants still using alcohol during the data collection period were labelled “Yes” and assigned the value code of 1. In addition, those who had stopped drinking alcohol during the data collection were labelled “No” and assigned the value code of 0.

Based on existing evidence on alcohol use, we classified the independent variables as socio-demographic (sex, age and grade), personal (not eating at restaurants, sedentary lifestyle and truancy), drugs and substance use (amphetamine use) and psychosocial (physical fight; physical attack; suicidal ideation, plan and attempt; bullied away from school, cyberbullied, parent’s/guardian’s use of tobacco, understanding parents, parental checks on completeness of assignment, multiple sexual partners, parent’s/guardian’s knowledge of what adolescent does with free time, loneliness and worry). The variables used in the analysis were labelled and coded as follows: age (1 = ≤15, 0 = ≥16), sex (1 = male, 0 = female), grade (1 = 1–3, 0 = 4–5), not eating at a restaurant (1 = yes, 0 = no), truancy (1 = yes, 0 = no), sedentary lifestyle (1 = yes, 0 = no) and amphetamines use (1 = yes, 0 = no). In addition, psychosocial factors were labelled and coded as follows: physical attack (1 = yes, 0 = no), physical fight (1 = yes, 0 = no), seriously injured (1 = yes, 0 = no), suicidal ideation (1 = yes, 0 = no), plan (1 = yes, 0 = no), attempt (1 = yes, 0 = no), bullied away from school (1 = yes, 0 = no), cyberbullied (1 = yes, 0 = no), parent/guardian use of tobacco (1 = yes, 0 = no) and understanding parents (1 = yes, 0 = no). Further, other variables were also coded and labelled: parent/guardian knowledge of what adolescent does with free time (1 = yes, 0 = no), parental/guardian checks on completeness of assignment (1 = yes, 0 = no), multiple sexual partners (1 = yes, 0 = no), loneliness (1 = yes, 0 = no) and worry (1 = yes, 0 = no). A decision to include any of these variables in the multivariate analysis was based on their conceptual relevance and statistical significance in the Chi-squared test.

### 2.5. Data Analysis

The data were analysed using IBM Statistical Package for Social Sciences (SPSS) version 27. A sample weighting approach of school, sex and students in each grade was used to represent school-going adolescents in Panama. The multiple imputations (MI) approach was used to replace missing values in the data, and the MI was applied in instances where the missing values exceeded 1%. The missing values allowed in our analysis were 1% to 10%, and those missing at random. Five MIs were conducted using the automatic imputation approach to preserve data quality in the presence of missing values. The observed values and the results using the complete case analysis were compared. A Pearson’s Chi-square test assessed the association between independent and dependent variables. Variables that were significant in the Chi-square test were included in a multiple binary logistic regression model. The results with their corresponding adjusted odds ratio (AOR) at a 95% confidence interval (CI) and level of significance set at *p* < 0.05 were reported.

## 3. Results

### 3.1. Background Characteristics of the Participants

**Figure 1:**
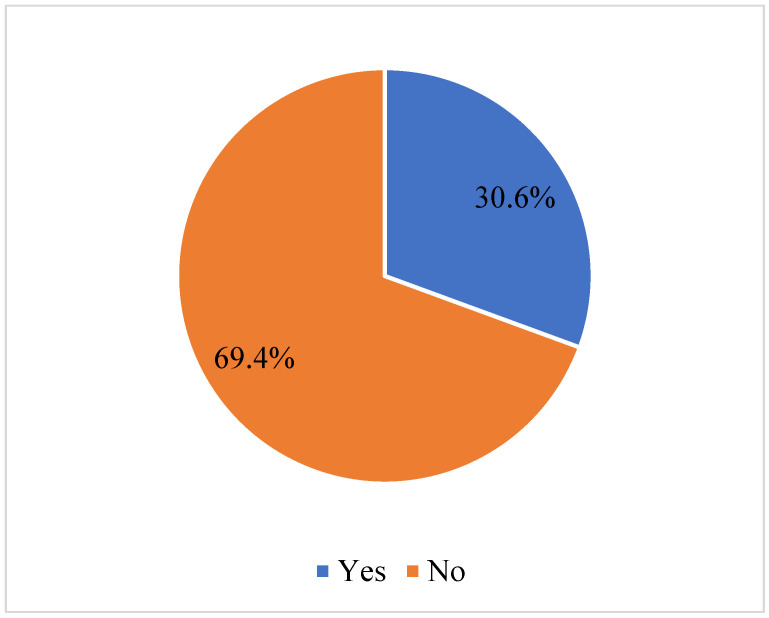
Prevalence of alcohol use among school-going adolescents in Panama.

The prevalence of alcohol use among participants was 892 (30.6%) (Figure 1). Significantly, 493 (16.9%) participants aged 16 years or above used alcohol. Further, 530 (18.2%) participants in grades 1–3 significantly used alcohol. Again, 303 (10.4%) participants did not eat from a restaurant, 173 (5.9%) of those were physically attacked, 254 (8.7%) of those engaged in physical fights and 481 (16.5%) of those who sustained serious injury significantly used alcohol. Further, 229 (7.9%) of participants who received suicidal thoughts, 186 (6.4%) of those who made suicide plans, 172 (5.9%) of those who attempted suicide and 186 (6.4%) of those who were bullied outside school significantly used alcohol. Also, significantly, 284 (9.7%) of truant adolescents, 194 (6.7%) of cyberbullied victims, 128 (4.4%) of those whose parents used any form of tobacco and 306 (10.5%) of those whose parents understood them used alcohol. Again, 539(18.5%) of participants engaged in a sedentary lifestyle, 312 (10.7%) of those whose parents checked whether they had completed their assignments and 314 (10.8%) of those who had multiple sexual partners significantly used alcohol. Further, 399 (13.7%) of those whose parents know what they do with their free time, 55 (1.9%) of those who use amphetamine, 184 (6.3%) of those who usually feel lonely and 131 (4.5%) of those who mostly worry significantly used alcohol (see Table 1).

### 3.2. Distribution and Chi-Square Test of Alcohol Use across Demographic, Psychological, Personal Attributes, Drug and Substance Use and Psychosocial Factors

The results show that demographic variables such as age (χ^2^ = 41.65, *p* < 0.001) and grade (χ^2^ = 47.13, *p* < 0.001) of participants were significantly associated with alcohol use. Further, personal characteristics such as not eating at a restaurant (χ^2^ = 72.650, *p* < 0.001), being truant (χ^2^ = 27.52, *p* < 0.001) and being sedentary (χ^2^ = 77.19, *p* < 0.001) were significantly associated with alcohol use. Further, psychosocial factors such as being a physically attacked victim (χ^2^ = 34.87, *p* < 0.001), engaging in physical fights (χ^2^ = 76.72, *p* < 0.001) and experiencing serious injury (χ^2^ = 41.13, *p* < 0.001) were significantly associated with alcohol use. In addition, having suicidal ideation (χ^2^ = 33.68, *p* < 0.001), planning (χ^2^ = 21.89, *p* < 0.001) and attempting suicide (χ^2^ = 23.92, *p* < 0.001) were significantly associated with alcohol use. Victims bullied outside school (χ^2^ = 34.57, *p* < 0.001), cyberbullied victims (χ^2^ = 34.84, *p* < 0.001), participants with parents who use tobacco (χ^2^ = 54.57, *p* < 0.001) and those whose parents understand them (χ^2^ = 16.44, *p* < 0.001) were significantly associated with alcohol use. Other psychosocial factors such as having parents who check whether an adolescent has completed their assignment (χ^2^ = 31.25, *p* < 0.001), those who had multiple sexual partners (χ^2^ = 199.87, *p* < 0.001), whose parents know what they do with their free time (χ^2^ = 33.99, *p* < 0.001), used amphetamines (χ^2^ = 16.41, *p* < 0.001), those who mostly feel lonely (χ^2^ = 20.56, *p* < 0.001) and those who feel worried (χ^2^ = 19.62, *p* < 0.001) were significantly associated with alcohol use (see Table 1).

### 3.3. Logistic Regression Analysis of Significant Factors Associated with Alcohol Use

Table 2 shows the binomial logistic regression results on the factors associated with alcohol use among adolescents in Panama. The odds of alcohol use were lower among adolescents in grades 1–3 than in grades 4–5 (AOR = 0.946, 95% CI = 0.538–0.781). In addition, the odds of consuming alcohol were lower among those who did not eat from a restaurant than those who ate from a restaurant (AOR = 0.589, 95% CI = 0.493–0.704). Again, the odds of alcohol use were significantly higher among those who engaged in a physical fight (AOR = 1.574, 95% CI = 1.260–1.965), were seriously injured (AOR = 1.362, 95% CI = 1.140–1.628), who mostly worried (AOR = 1.390, 95% CI = 1.055–1.832) and whose parents used any form of tobacco (AOR = 1.995, 95% CI = 1.491–2.67). Furthermore, the odds of alcohol use were higher among respondents who were sedentary (AOR = 1.771, 95% CI = 1.484–2.114), had multiple sexual partners (AOR = 2.845, 95% CI = 2.302–3.516) and who used amphetamines (AOR = 2.077, 95% CI = 1.226–3.522) (see Table 2).

## 4. Discussion

Alcohol consumption is a global public health phenomenon, but the consequences are dire when adolescents are initiated into early consumption. This present study investigated the prevalence and predictors of alcohol use among school-going-age adolescents in Panama. The findings showed that the prevalence of alcohol use among adolescents was 30.6%. This outcome is of a public health concern as any prevalence of alcohol usage among a vulnerable demographic group, such as adolescents, spells doom for the future public health of Panama and its economic workforce. Comparatively, the current finding is higher (30.6%) than previous studies in Thailand, 14.8% [35], Malaysia, 8.9% [18], India, 15% [25], the Eastern Mediterranean region, 10%, and Africa, 29.3% [36].

Conversely, extant literature has reported a higher prevalence of alcohol use than the present study. For example, the WHO estimated prevalence of alcohol use among adolescents aged 15–19 at 52.7% in the Americas and 69.5% in Europe [36]. In comparison, a systematic review by Nadkarni et al. [24] in India reported a range from 3.9% to 69.8% of prevalence. Many reasons could be inferred for the growing trend of alcohol use among adolescents in the study population and the variations observed across studies. Foremost, adolescents may resort to alcohol usage due to peer influence, bullying and abuse by adults, parents’ subtle introduction and poor parenting styles. In addition, adolescents may be using alcohol to resolve pain, conceal self-failures and other socially undesirable acts, which Jessor and Jessor [5] explained in the problem behaviour theory. This theory indicated that adolescents who adopted certain behaviours that gave them independence and autonomy from family and gained them the acceptance of peers were also at risk of social vices, including alcohol use. Therefore, health promotion stakeholders must understand the driving factors in the adolescent stage to institute age-appropriate interventions that may help curb alcohol use [4,28].

Additionally, several socio-demographic variables such as access to disposable income [37], neighbourhood socio-economic status [38] and the legal age of alcohol initiation may account for the prevalence of alcohol use and the variation in prevalence amidst other methodological differences. Besides these pathways, cross-national differences in alcohol use have been linked to increased globalisation, industrialisation, urbanisation and mass media exposure; this linkage may be because of how these factors affect traditional family and community controls on the transition to adulthood, such as diminished parental control, social support and social cohesion, as well as the quick spread of non-traditional ideas and values [29,30,31,39]. There is also the unrestricted marketing of harmful behaviours and lifestyles by multinational cigarette and alcohol businesses in low- and middle-income nations [40] targeted at young people [41]. We suggest that the Panamanian Food Safety Authority and media commission tackle and address such unwholesome products targeted at young children. Fortunately, some studies have unravelled some protective factors in some settings that may not be the panacea but may help stem the growing culture of alcohol use. For instance, religious beliefs as well as Africentric values such as collectivism, self-determinism and purpose [42,43] are but a few protective factors that may mitigate the alarming rate of alcohol use among adolescents.

Furthermore, the odds of alcohol use were high among sedentary respondents with multiple sexual partners and those who used amphetamines. These triad factors, alcohol usage, multiple sexual partners and amphetamines usage, are negative risky behaviours boosted by sedentarism [44,45,46,47,48]. Though the literature within Panama on the subject is sparse, the extant literature on multiple sexual partners and alcohol consumption agrees with our findings. For instance, studies by Choudhry et al. [49], DiGrande et al. [50] and Santelli et al. [51] found an association between alcohol use and multiple sexual partnerships among adolescents. The link between alcohol use and sexual relationship has been established in many studies. Alcohol use may increase sexual risk-taking through behavioural and biological factors [52]. This is due to the pharmacological effects of alcohol on cognitive abilities, which disinhibit behaviour, according to the alcohol myopia theory [52]. As a result, proximal and simple cues that initiate activity, such as sexual excitement, are still processed, while more distal and complex cues that would typically limit behaviour, such as the potential of getting an STI or becoming pregnant unexpectedly, are no longer sufficiently processed. This finding reiterates the risky behaviours of adolescents that continue to increase HIV/AIDS and other related sexually transmitted infections. The Ministry of Social Development should work with other youth agencies to strengthen physical activity and reduce sedentarism, as that has been linked to alcohol use and other vices such as multiple sexual partner relationships [48]. This connection is drawn from Bernabe-Ortiz and Carrillo-Larco’s [53] findings that sedentarism among the youth of Latin America and the Caribbean regions, where Panama belongs, appeared to be higher (≥40%) coupled with being physically inactive (≥20%). This assertion may be one of the reasons for alcohol use, among others. Encouraging youth in physical activities will engage more of their time in productive education and positive social network development. Also, misinformation concerning alcohol as possessing aphrodisiac capabilities to boost sexual pleasure should be addressed through health promotion activities.

Again, the odds of alcohol use were significantly high among those who engaged in physical fights, were seriously injured, and whose parents used any form of tobacco. This outcome is similar to prior findings, where the odds of sustaining injuries were high among adolescents who drink alcohol [35,54,55,56,57]. According to these earlier studies, adolescents who use drugs or alcohol are more likely to sustain significant injuries, which may be deliberate or unintentional. The plausible reason for this assertion may be due to the impaired judgement as a result of the pharmacological effects of alcohol on the cognitive synthesis of danger versus non-danger, thus exposing many adolescents to risky behaviours. Further, adolescents who abused or misused alcohol were more likely to engage in physical attacks and be bullied outside school. This finding supports previous studies [54,57,58,59,60,61]. For example, a longitudinal study by Wialliam et al. [61] found a prospective association between alcohol use, physical attacks and bullying among in-school adolescents. However, our study found no significant predictive association between bullying and alcohol use.

Notwithstanding, school public health nurses are one possible solution to this since bullying victims have been shown to benefit from seeing a school health nurse [62,63], and schools may be able to minimise perpetration as well. The observed similarities in the findings may signal the cross-cultural and wide-ranging nature of adolescent indulgence in alcohol use. Additionally, technology and urbanisation have bridged knowledge gaps and information transfer as adolescents use various media platforms, which are becoming common advertisement platforms by alcohol producers [64]. Schools should also institute preventive interventions (e.g., positive behavioural strategies such as rational emotive behavioural education, peer educator network systems, face-to-face counselling sessions, and alcohol/substance use cessation therapy) that are fundamental in promoting a positive school climate that might help reduce adolescents’ alcohol use and, perhaps, other anti-social behaviours (e.g., physical fights, bullying, suicidal ideation and attempts).

We further found that adolescents who were worried were more likely to abuse alcohol compared with their peers who did not worry. Worrying is a defining hallmark of several internalising disorders, including anxiety and depression. Previous studies have explained the role of anxiety and depression in alcohol-use behaviours [65,66]. Among school-going adolescents, several reasons, including but not limited to low academic performance, class repetition and lack of acceptance among peer groups, may be major sources of worry and concern for them. It is plausible to argue that these reasons and many others may serve as key precipitants in the internalising-to-alcohol-use pathway among school-going adolescents in Panama. School psychologists may play an instrumental role in helping students overcome situations that may trigger various forms of worry. Thus, Panamanian school authorities need to integrate the services of school-based psychologists to help these adolescents to deal effectively with worrying situations and subsequently reduce the likelihood of alcohol use among them.

### Strengths and Limitations

This study has the potential to advance practices for preventing alcohol use and multiple sexual partners, sedentary lifestyles and physical attacks in high schools in Panama, as well as revealing the critical role parents can play in reducing alcohol use and its associated vices among adolescence. Notwithstanding the preceding strengths, the following limitations should guide the use of our findings. There are no discernible cause-and-effect linkages in this cross-sectional investigation. This study was restricted to participants aged 13 to 17 years and cannot be applied to other demographics. This study also uses self-report measures, meaning that some participants may have provided socially acceptable answers, leading to under- and overreporting of their alcohol behaviour. Another limitation is that we did not investigate the frequency of usage or use at a later time-point, such as the previous year. Also, utilising single items to measure psychosocial variables such as alcohol use and suicidal behaviours among participants may not be sufficient to obtain the adequate state of behaviours.

## 5. Conclusions

The current study’s results should be considered when developing and carrying out alcohol prevention programmes for adolescents. First, stakeholders within the Education Ministry should implement age-appropriate alcohol prevention interventions, especially among high-grade adolescents. Further, clinical psychologists and other mental health workers should be used in schools to offer counselling and therapy to physically abused students and those who worry. This study found that lacking supportive parenting practices, such as rarely or never assisting children with homework, increased current alcohol consumption and binge drinking in adolescents, particularly in the higher-grade category. These findings suggest that parental involvement in alcohol use prevention programmes is essential. Sedentarism was also associated with alcohol use in our study. The MoH and the MoE are encouraged to institute policies for youth through physical education, exercise and sports to help spur physical activity among school-going adolescents.

## Figures and Tables

**Table 1:** Association of risk factors with suicidal ideation, plan and attempts among respondents.

Variables		Alcohol Use			*φ* *_c_*
		Yes	No	Chi-Square (χ^2^)	
Demographic					
Age (years)	≤15	399 (13.7%)	1166 (40.0%)	41.65 ***	−0.120
	≥16	493 (16.9%)	856 (29.4%)		
Sex	Male	425 (14.6%)	890 (30.5%)	3.29	0.034
	Female	467 (16.0%)	1132 (38.8%)		
Grade	1–3	530 (18.2%)	1461 (50.1%)	47.13 ***	−0.127
	4–5	362 (12.4%)	561 (19.3%)		
Personal					
Not eating at a restaurant	Yes	303 (10.4%)	1032 (35.4%)	72.650 ***	−0.158
	No	589 (20%)	990 (34.4%)		
Truancy	Yes	284 (9.7%)	458 (15.7%)	27.52 ***	0.097
	No	608 (20%)	1564 (53.7%)		
Spent three hours sitting	Yes	539 (18.5%)	865 (29.7%)	77.19 ***	0.163
	No	353 (12.1%)	1157 (39.7%)		
Psychosocial					
Physically attacked	Yes	173 (5.9%)	227 (7.8%)	34.87 ***	0.109
	No	719 (24.7%)	1795 (61.6%)		
Physical fight	Yes	254 (8.7%)	297 (10.2%)	76.72 ***	0.162
	No	638 (21.9%)	1725 (59.2%)		
Seriously injured	Yes	481 (16.5%)	831 (28.5%)	41.13 ***	0.119
	No	411 (14.1%)	1191 (40.9%)		
Suicidal thought	Yes	229 (7.9%)	333 (11.4%)	33.68 ***	0.108
	No	663 (22.8%)	1689 (58.0%)		
Made a plan	Yes	186 (6.4%)	282 (9.7%)	21.89 ***	210.893
	No	706 (24.2%)	1740 (59.7%)		
Attempted suicide	Yes	172 (5.9%)	250 (8.6%)	23.92 ***	0.091
	No	720 (24.7%)	1772 (60.8%)		
Bullied off school	Yes	186 (6.4%)	251 (8.6%)	34.57 ***	0.109
	No	706 (24.2%)	1771 (60.8%)		
Cyberbullied	Yes	194 (6.7%)	265 (9.1%)	34.84 ***	0.109
	No	698 (24.0%)	1757 (60.3%)		
Parents’/guardians’ use of tobacco	Yes	128 (4.4%)	222 (4.2%)	54.57 ***	0.137
	No	764 (26.2%)	1900 (65.2%)		
Understanding parents	Yes	306 (10.5%)	855 (29.3%)	16.44 ***	−0.075
	No	586 (20.1%)	1167 (40.0%)		
Parents/guardians check the completeness of assignment	Yes	312 (10.7%)	932 (32.0%)	31.25 ***	−0.104
	No	580 (19.9%)	1090 (37.4%)		
Multiple sexual partners	Yes	314 (10.8%)	256 (8.8%)	199.87 ***	0.262
	No	578 (19.8%)	1766 (60.6%)		
Parents/guardians knowledge of what adolescent does with his/her free time	Yes	399 (13.7%)	1141 (39.2%)	33.99 ***	−0.108
	No	493 (16.9%)	881 (30.2%)		
Amphetamine use	Male	55 (1.9%)	1260 (43.2%)	16.41 ***	0.112
	Female	27 (0.9%)	1572 (53.9%)		
Loneliness	Yes	184 (6.3%)	282 (9.7%)	20.56 ***	0.084
	No	708 (24.3%)	1740 (59.7%)		
Worried	Yes	131 (4.5%)	185 (6.3%)	19.62 ***	0.082
	No	761 (26.1%)	1837 (63.0%)		

Note: *** *p* < 0.001.

**Table 2:** Relationship between the significant variables and alcohol use among adolescents.

Variables	B	Wald Test (z-Ratio)	Adjusted Odds Ratio	95% Confidence Interval for the Odds Ratio	
				Lower	Upper
Demographic					
Sex (male)	−0.055	0.350	0.946	0.787	1.137
Age	−0.027	0.044	0.973	0.754	1.257
Grade	−0.434 ***	20.659	0.648	0.538	0.781
Personal					
Did not eat food from a fast food restaurant	−0.529 ***	33.953	0.589	0.493	0.704
Truancy	0.142	2.027	1.153	0.948	1.403
Sedentary lifestyle	0.571 ***	40.092	1.771	1.484	2.114
Drug/substance use					
Amphetamine use	0.731 **	7.372	2.077	1.226	3.522
Psychosocial					
Physically attacked	0.188	2.056	1.206	0.933	1.559
Physical fight	0.454 ***	16.003	1.574	1.260	1.965
Seriously injured	0.309 **	11.540	1.362	1.140	1.628
Suicidal ideation	0.139	0.945	1.149	0.868	1.520
Suicidal plan	−0.035	0.052	0.966	0.717	1.301
Attempted suicide	0.021	0.020	1.022	0.763	1.368
Bullied off campus	0.188	2.197	1.206	0.941	1.546
Cyberbullied	0.194	2.444	1.214	0.952	1.547
Parental use of tobacco	0.691 ***	21.580	1.995	1.491	2.670
Understanding parents	0.074	0.491	1.077	0.876	1.324
Parents check on the completion of assignments	−0.162	2.591	0.851	0.698	1.036
Multiple sexual partners	1.046 ***	93.603	2.845	2.302	3.516
Parental knowledge of what adolescent does with free time	−0.078	0.614	0.925	0.761	1.124
Loneliness	0.076	0.346	1.079	0.838	1.389
Worry	0.330 *	5.480	1.390	1.055	1.832
Constant	−6.434	61.689	0.002		

Note. * *p* < 0.05, ** *p* < 0.01, *** *p* < 0.001; Hosmer and Lemeshow test (goodness of fit), χ^2^ (8) = 4.649, *p* = 0.794. The World Bank Group. Total alcohol consumption per capita male (litres of pure alcohol, projected estimates, male 15+ years of age). Retrieved from: https://databank.worldbank.org/rports.aspx?source=2&sries=SH.ALC.PCAP.MA.LI&country=PAN# (accessed on 29 March 2023).

## Data Availability

The dataset used for this analysis for this study was obtained from the GSHS–Panama, 2018. Access to the data can be obtained at the WHO website: https://extranet.who.int/ncdsmicrodata/index.php/catalog/879 (accessed on 5 March 2023).
